# A comparative study on the chloride effectiveness of synthetic rutile and natural rutile manufactured from ilmenite ore

**DOI:** 10.1038/s41598-021-83485-6

**Published:** 2021-02-17

**Authors:** Eun Jin Jung, Jinyoung Kim, Ye Rin Lee

**Affiliations:** 1 Research Institute of Industrial Science and Technology (RIST), 67 Cheongam-ro, Nam-gu, Pohang, 37673 Republic of Korea; 2 Korea Metal Material Research Association, 135, Jungdae-ro, Songpa-gu, Seoul, 05717 Republic of Korea

**Keywords:** Solid Earth sciences, Chemistry, Energy science and technology, Materials science

## Abstract

Studies on continuous and selective chlorination by using ilmenite have been actively conducted because the efficient removal of FeO from ilmenite(FeTiO_3_) ore using selective chlorination not only improves the reaction purity of TiCl_4_ but it also leads to price competitiveness compared to TiCl_4_ synthesized from natural rutile. The chlorination of synthetic rutile with FeO removed was compared with that of natural rutile to examine the reaction efficiency. The selective chlorination efficiency depends on the input amounts of coke and Cl_2_, as shown by thermodynamic calculation, when FeO is selectively removed. It was found that manufacturing of TiCl_4_ was easier by using the synthetic rutile, because it had greater porosity than natural rutile. Relatively greater pore volumes were found in the synthetic rutile than in natural rutile. It was confirmed that the reaction efficiency of chlorination for TiCl_4_ production was directly related to the difference in the porosity distribution between the titanium ores, as verified by a kinetic comparison of synthetic and natural rutiles.

## Introduction

Ilmenite(FeTiO_3_) ore is a raw material for producing TiCl_4_ through the chlorination method and the sulfuric acid reaction method. TiCl_4_ is used as a raw material for producing metallic titanium through the Kroll process or for producing TiO_2_ used as a catalyst or pigment^1–5^. Until now, high-grade rutile ore has been used for manufacturing TiCl_4_. The advantages of having low prices and extensive reserves have led to active research using the ilmenite ore. To produce TiCl_4_ using ilmenite ore, it is important to secure high-quality TiO_2_ by removing FeO from the ore^6–8^. Hydrometallurgical methods to produce TiCl_4_ include the Ishihara method that uses sulfuric acid, the Benilite and Murso methods that uses hydrochloric acid^9^, and the Becher method that uses NH_4_Cl + HCl^10^. Sneha Samal^11,12^ evaluated that the titania slag is dissolved by using ethylene glycol or resorcinol. The upgraded feed stock was obtained by control of acid concentration, pulp density, grain size and time. Alternatively, Paliyaguru et al.^13^ recently manufactured TiO_2_ nanoparticles with gravity separation properties using H_3_PO_4_ acid in ilmenite ore. Wu et al.^14^ manufactured TiO_2_ with hydrolysis of TiCl_4_ using AlCl_3_. This makes use of rutile's high chromium absorption capacity, which also helps to solve water pollution. Ma et al.^15^ evaluated the efficiency of application of titanium industries through structural evaluation using viscous properties of high titanium slag. In this way, research on the recovery of titanium through wet reactions is active in trying a different reaction method than in the past. In addition, the dry smelting method in titanium research has the advantage of having fewer by-products compared to wet smelting methods. Therefore, the active progress of researchers on reaction testing is meaningful in order to increase the industrial application efficiency of titanium.

As a dry method, upgrade slag (UGS) can be obtained by separating slag containing TiO_2_ from pig iron through reduction of FeO_x_ in an electric furnace^16^; however, reducing the FeO from the ilmenite ore at 1550–1600 °C causes the gradually concentrated TiO_2_ to lose fluidity due to its high melting point, so there is a limit to obtaining high purity TiO_2_.

Producing TiCl_4_ using ilmenite ore is more economical than using natural rutile because the price of ilmenite is approximately four times lower^17^. However, to produce TiCl_4_ from ilmenite ore using selective chlorination, technical factors must also be examined. These include the roasting process for the phase control of FeO_x_ present in the ore, the derivation of high-purity TiO_2_ production conditions for the selective chlorination of FeO. The synthetic rutile fabricated through the selective chlorination of FeO may have large benefits in terms of the reaction efficiency because its porosity is higher than that of natural rutile.

Synthetic rutile refers to TiO_2_ that remains in the ilmenite after FeO is removed. Synthetic rutile is more porous than natural rutile, owing to the removal of FeO from the matrix. To identify an appropriate chlorination process, it is essential to compare reactivity through a quantitative evaluation of the porosity and reactive efficiency by rutile species. Furthermore, the observation of morphology is essential to evaluate the reaction efficiency of chlorine reactions in synthetic/natural rutile. As part of this review, Li et al.^18^ conducted structural considerations on the reaction with XRD and FT-IR in the process of recovering vanadium and chromium through microwave absorption characteristics and thermal behavior evaluation of vanadium slag. Chen et al.^19^ recovered Cr, V using sodium carbonate roasting for high-purity shuttle production. We consider the analysis of phase changes through XRD, SEM, and Raman analyses. Kang et al.^20^ also considered the behavior of rutile and sodium carbonate reactions in the microwave heating reaction through structural evaluation. Thus, morphology evaluation by structural analysis are essential factors in assessing the chemical reaction. In this study, the FeO with selective chlorination by using coke and Cl_2_ gas of ilmenite ore was eliminated, and TiO_2_ of high purity produced. The reaction characteristics based on changes in porosity were investigated by comparing the efficiency of the chloride reaction with natural rutile of high purity.

## Material and methods

The ilmenite ore (Shijiazhuang Lanhu Welding Consumables Co., Ltd., China) and coke (Fushun Fangda High Tech Material Co., Ltd., China, 0.03wt% FeO_x_-0.09wt% SiO_2_-0.04wt% Al_2_O_3_-99.36wt% C-0.48wt% S) were prepared for the synthetic rutile manufacturing.

The average diameter of ilmenite ore and coke was 214 and 803 μm, respectively. And the synthetic rutile was produced through the reaction with chloride gas. To exclude reaction and influence of moisture during chlorination process, ilmenite and coke were first dried in a furnace at 150 °C for 24 h. A fluidized bed reactor(FBR) for chloride process was used. The inner dimeter of the FBR is 2.54 cm, and the tube is 70 cm high. We used 46.73 g of ilmenite ore, which met our length to diameter requirement (L/D = 3).

The coke was injected by relying on the stoichiometry ratio of FeO contained in the ilmenite ore.

Thus, FeO in ilmenite and coke were maintained at a ratio of 1:1. The 3.70 g of coke was added to meet the 1:1 M ratio for FeO_x_ in ilmenite and coke to suit selective chlorination. The coke used in the experiment was assumed to be all carbon because of high purity coke (contains more than 99% carbon composition).

The reaction temperature was set to 1173 K and it was increased at a rate of approximately 15 K/min; nitrogen was continuously injected at a rate of 300 ml/min to maintain fluidization of the ore. When the set temperature was reached, the inputted nitrogen gas was stopped. Chlorine gas was injected at a flow rate of 851 ml/min for the selective chlorination process. The minimum fluidization velocity was calculated using the Wen&Yu equation^21^, and the minimum fluidization velocity for each ilmenite and coke was calculated as the ratio of the raw material. Experimentally confirmed that the minimum fluidization velocity should be approximately twice through the cold test, and if it is higher than twice, the ilmenite and coke will be separated, and if it is lower than twice, the injection material will not be mixed. Therefore, the flow velocity could be twice the minimum fluidization velocity. To compare the chlorination behavior under the same FeO content included in natural rutile, the selective chlorination time was controlled to 40 min for a preliminary test. The synthetic rutile, which was produced through the chlorination of ilmenite ore, and natural rutile ore (Vol'nogorsk Mining, Ukraine) were mixed with coke for 30 min to observe the reaction surface after chlorination process. The particle sizes of the two rutile types ranged from 180 to 210 µm. After the stoichiometric mixture (rutile:coke = 1:2 mol) was left for 30 min for reaction, the supply of Cl_2_ at 1173 K was stopped and Ar gas was inputted into the reaction tube to remove the remaining Cl_2_ gas. The TiCl_4_ was acquired by a temperature controlled receiver with cooling water. The discharged unreacted Cl_2_ gas was sent into NaOH aqueous solution to be neutralized and then discharged to the atmosphere.

The selective chlorination of FeO from FeTiO_3_ ore is performed according to the following process in Eq. (1). The following reaction confirmed that selective removal was completed after 40 min of chlorination. Information of composition on the types of ore is given in Table 1.

1 $${\text{FeTiO}}_{{3}} + {\text{C}} + {1}.{\text{5Cl}}_{{2}} \to {\text{FeCl}}_{{3}} + {\text{TiO}}_{{2}} + {\text{CO}}$$

**Table 1:** Chemical compositions of ilmenite, synthetic rutile, and natural rutile (wt%).

Content (wt%)	TiO_2_	FeO_x_	SiO_2_	Al_2_O_3_	Cr_2_O_3_	MgO	MnO	CaO
Ilmenite	53.59	36.9	3.41	1.97	1.00	0.90	1.23	0.20
*Synthetic rutile	86.7	2.08	4.69	2.22	1.07	0.86	0.05	0.27
Natural rutile	92.5	1.65	2.47	1.01	0.13	0.11	0.06	0.05

*Synthetic rutile: obtained by selective chlorination from ilmenite (after 40 min at 1173 K).

Later, TiCl_4_ was produced from the synthetic and natural rutile through the following chemical reaction in Eq. (2). After the reaction, the reaction efficiency was compared by measuring the weight of the residue in Eq. (3).

2 $${\text{TiO}}_{{2}} + {\text{2C}} + {\text{2Cl}}_{{2}} \to {\text{TiCl}}_{{4}} + {\text{2CO}}$$

3 $$\mathrm{Reaction efficiency }\left(\mathrm{\%}\right)= \frac{{m}_{0}-m}{{m}_{0}}\times 100$$

(m_0_ : initial mass of sample, m : mass of residue).

After completion of the experiment, the remaining reactants were collected and weighed, and then the surface of the residue was observed using scanning electron microscopy (SEM, JEOL, JSM-6610LV). Later, the porosities of the ores were measured using a specific surface area analyzer (Tristar II 3020, Micromeritics) to compare the synthetic rutile with the natural rutile. The microstructures of the ores were observed using transmission electron microscopy (TEM, FEI Tecnai OSIRIS).

## Results and discussion

**Figure 1:**
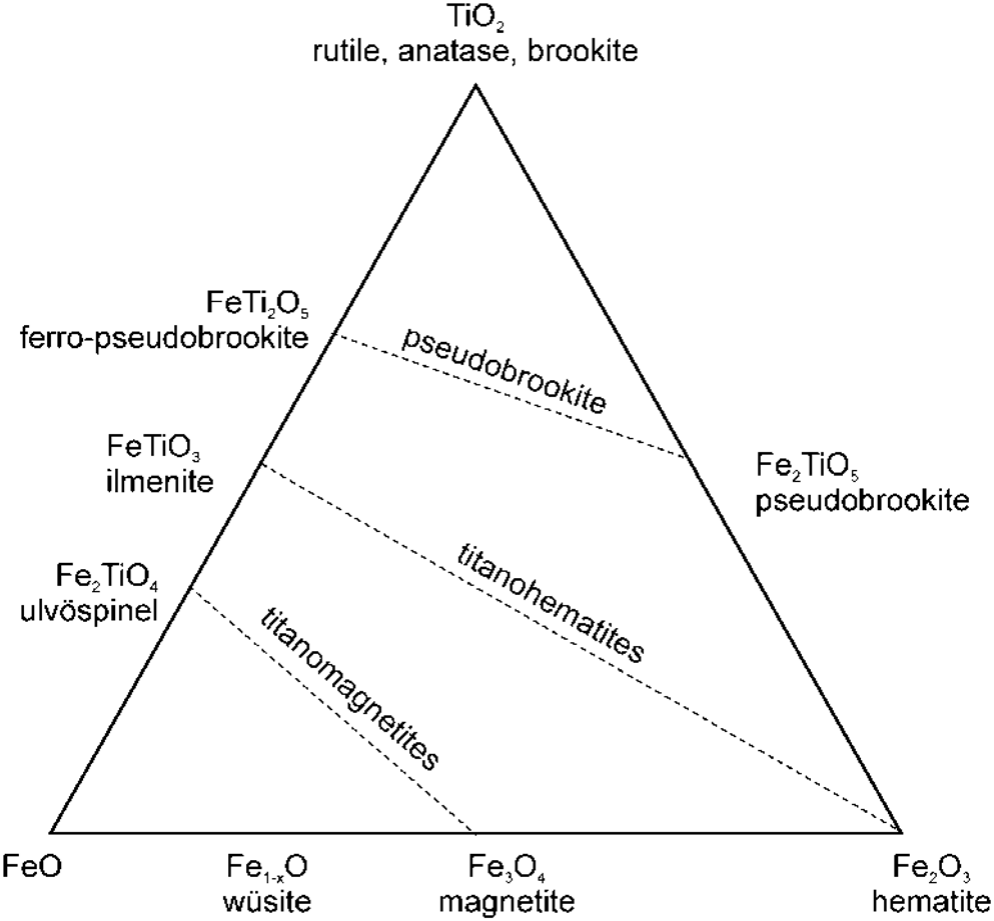
FeO-Fe_2_O_3_-TiO_2_ ternary system.

Figure 1 shows the phase diagram of FeO-Fe2O3-TiO2 by Meinhold^22^, and I redrew it again. It shows that the phase can vary depending on each stoichiometric state. Phases such as FeO, Fe_2_O_3_, TiO_2_, and Fe_2_TiO_5_, in addition to FeTiO_3_, can exist together for ilmenite ore. The presence of such multiple phases not only lowers the reaction efficiency but also makes it difficult to realize selective chlorination due to the existence of phases, such as Fe_3_O_4_ that is particularly difficult to reduce. For this reason, many researchers have performed chlorination after roasting the ilmenite to increase the reaction efficiency. The FeO in ilmenite (FeTiO_3_) is theoretically FeO; however, the actual phase shows that the ore contains FeO, Fe_2_O_3_, Fe_3_O_4_, and so on, making it a difficult reduction condition. The order of reduction efficiency is as follows: FeO > Fe_2_O_3_ > Fe_3_O_4_; This is determinated to be due to volumetric expansion changes depending on the difference in the crystal structure when each oxide reacts with the reducing agent^23–26^.

Most of these are mixed with FeO and Fe_2_O_3_ and become Fe_3_O_4_. The chlorination reaction of Fe_3_O_4_ phase reduces the removal efficiency from ilmenite ore. Therefore, it becomes an issue because the efficiency of the roasting process for controlling the FeO_x_ phase and the process time accordingly depend on selective chlorination of FeO in ilmenite ore.

Gan et al. examined the oxidation path by roasting ilmenite ore and reported that the path varied depending on the oxidation roasting conditions of below 825 °C or above 950 °C^27^. Fu et al. controlled the temperature, particle size, and oxygen partial pressure of ilmenite ore and observed the phase transitions for reaction mechanism of ilmenite oxidation^28^. They reported that ilmenite ore can exist as TiO_2_, Fe_2_O_3_, Fe_2_Ti_3_O_9_, and Fe_2_TiO_5_ depending on the reactor conditions. Allen observed the magnetic properties of ilmenite using magnetic attraction and rotation separation and derived the conditions of magnetic rotation separation through the particle rotation of the ilmenite sample at each temperature while the temperature was varied from 450 to 650 °C^29^. As the chlorination efficiency actually depends on the remaining magnetic oxides in ilmenite, iron oxides can be pretreated in the form of hematite.

As mentioned, many researchers have reviewed the FeO_x_ phase control in the ilmenite ore and thus can acquire efficient synthetic rutile. In addition, even if the phase of the FeO_x_ is controlled, reaction behavior may vary depending on the ratio of the coke and chlorine gas injected with the ilmenite ore. Thus, iron chloride is made at FeCl_2_ and FeCl_3_, depending on the amount of chlorine gas injected, and relatively large coke and chlorine injections may not be selective chloride. The reaction formula for the injection of coke and chlorine was shown in Eqs. (1), (4) and (5).

4 $${\text{FeTiO}}_{{3}} + {\text{C}} + {\text{Cl}}_{{2}} \to {\text{FeCl}}_{{2}} + {\text{TiO}}_{{2}} + {\text{CO}}$$

5 $${\text{FeTiO}}_{{3}} + {\text{3C}} + {3}.{\text{5Cl}}_{{2}} \to {\text{FeCl}}_{{3}} + {\text{TiCl}}_{{4}} + {\text{3CO}}$$

When FeTiO_3_ was inputted, the synthetic rutile made by the chlorination of the mixture of the ore and coke were obtained.

**Figure 2:**
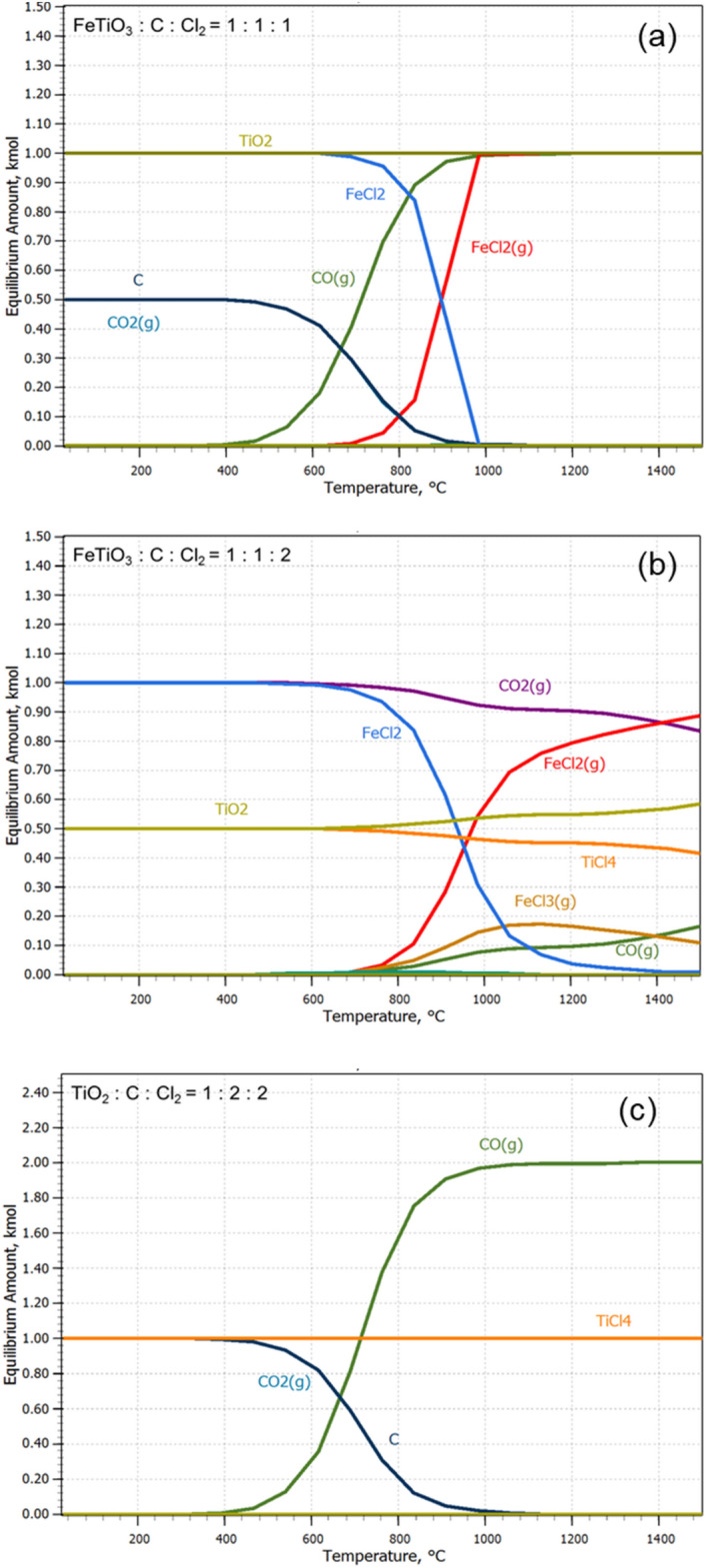
Equilibrium state of ilmenite and rutile ore with varying input amounts of coke and chloride gas.

Thermodynamic calculation using HSC Chemistry v.9.0 software as inputted materials is shown in Fig. 2. HSC chemistry is a software that can thermodynamically identify the stabilization of responses under a given condition by calculating Gibb’s free energy. Figure 2a,b show that different products were generated depending on the stoichiometric input of FeTiO_3_, C, and Cl_2_, and the optimal conditions for generation of FeCl_2_ by selective chlorination and the mixed reaction up to TiCl_4_ were determined. If the inputted FeTiO_3_, C, and Cl_2_ are reactivated within a given temperature range, the stabilization phase can be verified, and optionally the conditions for removing FeO can be determined. As indicated by the results, this depends on the injection mole ratio of the reducing agent (coke) and Cl_2_ gas, and not the main factor for temperature control in the operation process. Depending on the injection mole ratio, the loss of TiO_2_ may or may not occur at the same time as the FeO is removed. As shown in Fig. 2, the progress temperature for removing FeO exceeded 900 °C, and ferrous chloride was gasified.

**Figure 3:**
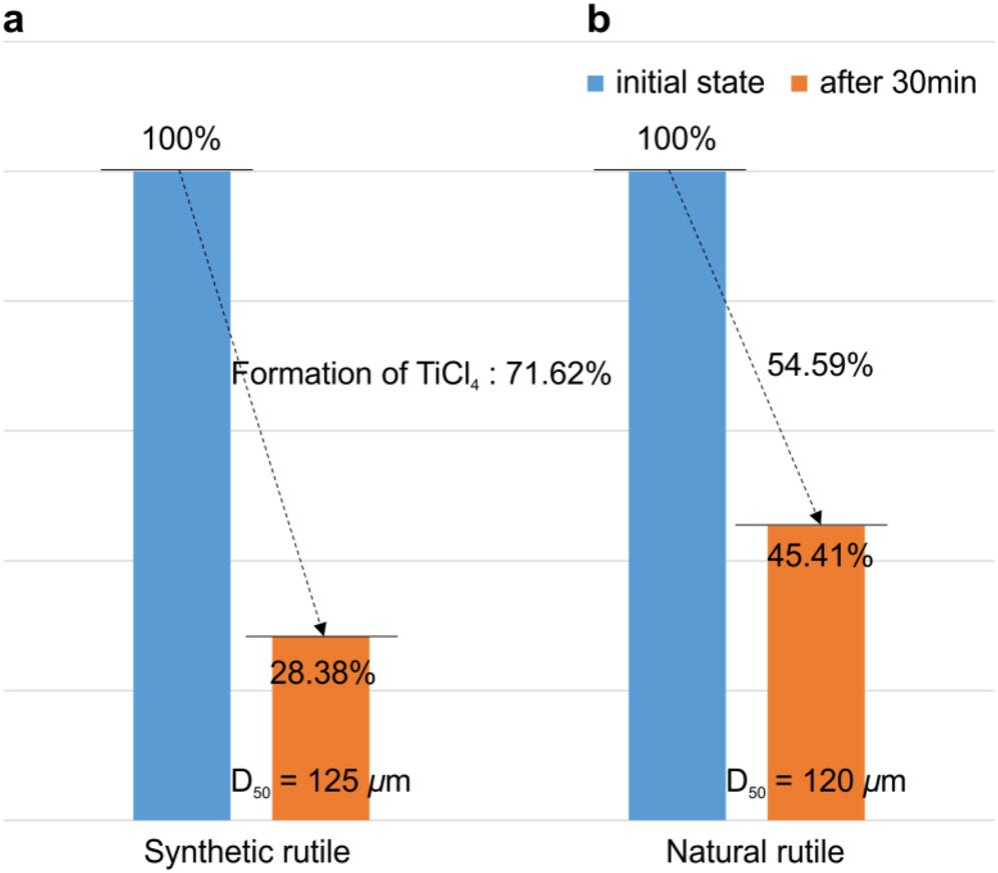
Weight change rate of synthetic rutile and natural rutile after chlorination.

Kang et al. examined the conditions for deriving selective phases through the control of the chlorine partial pressure, oxygen partial pressure, and temperature^30,31^. They stated that the selective chlorination of FeCl_2_ was possible when log *P*_Cl2_ was controlled between − 10 and 0 atm at 1100 K and that it is important to fabricate TiCl_4_ using high-purity synthetic rutile residue after selective chlorination. Actually, the generated phase was different depending on the partial pressure of the chlorine. As this is directly related to the purity of the product and synthetic rutile residue, the control of the input amounts is very important. As shown in Fig. 2c, TiCl_4_ can be produced through the chlorination of rutile with coke and chlorine, and the reaction efficiency can be examined through the thermodynamic driving force. Although the synthetic rutile and natural rutile have the same chemical formula and crystal structure, they have different internal microstructure, resulting in different reaction efficiency. In the case of the synthetic rutile, the initial FeO was selectively chlorinated and removed as FeCl_2_ or FeCl_3_, resulting in increased porosity; this porosity facilitates chlorine penetration. Figure 3 shows the reaction efficiency of the synthetic and natural rutile after chlorination for 30 min. Swanepoel observed the change in the shell reaction considering the shrinking-core model for the pore diffusion in the ore by hydrochloric acid^32^. The reaction efficiency was examined by kinetic considerations of temperature, particle size, and molar ratios of the synthetic rutile (Fig. 3a) and natural rutile (Fig. 3b), as shown in Fig. 3. Synthetic rutile were manufactured by chloride reactions from the ilmenite ore, and the particle size of the synthetic rutile was 125 µm after a 30-min chloride reaction. The natural rutile is 120 µm, but it means that the difference in particle size in the evaluation of the chloride reactions of two types of rutiles has been ignored. After chlorination process, there was a difference in the reaction efficiency. This appears to be because of the difference in the internal porosity.

**Figure 4:**
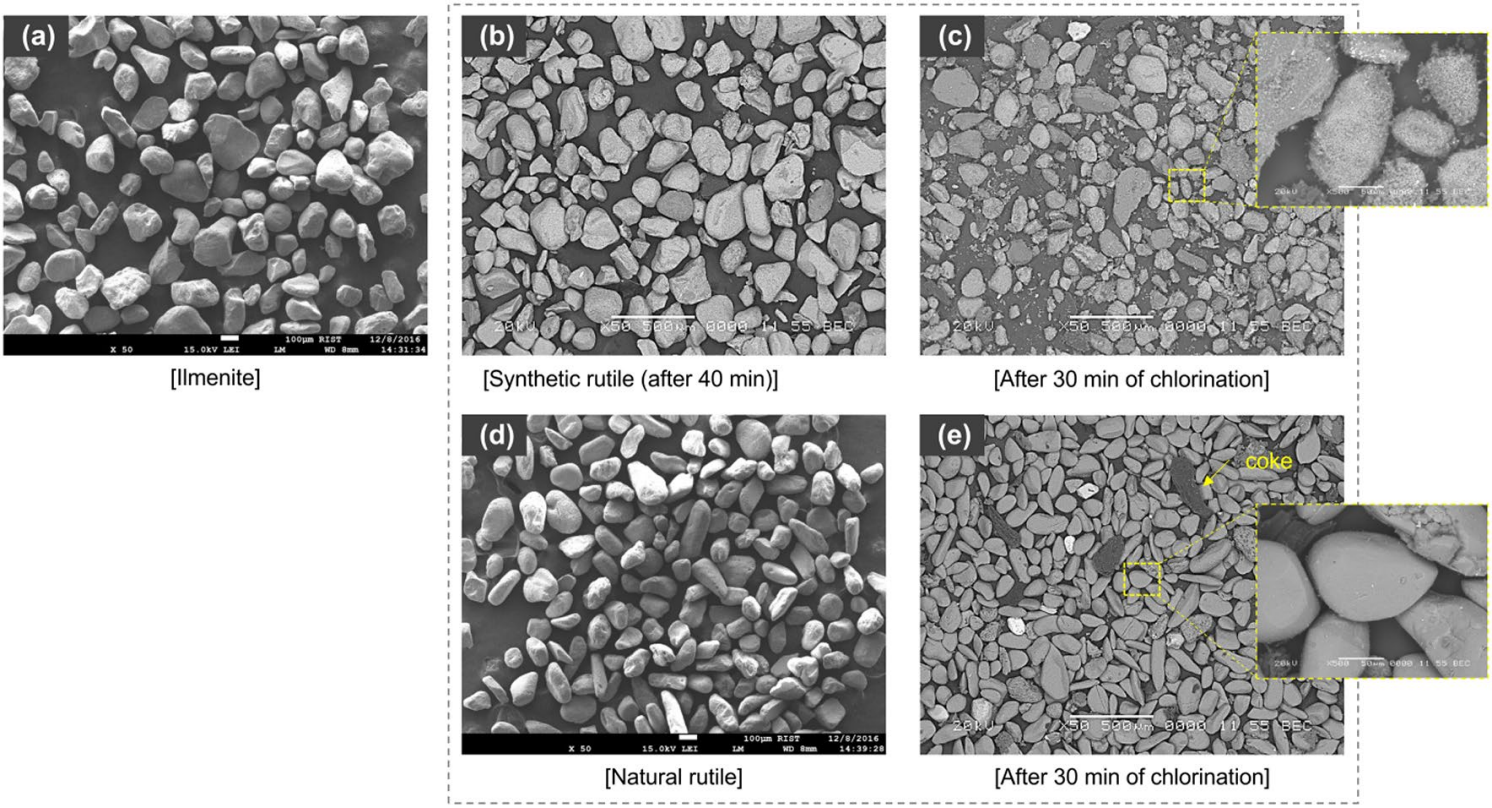
SEM images of surface morphology for (**a**) raw ilmenite and (**b**) ilmenite chlorinated for 40 min (**c**) synthetic rutile chlorinated for 30 min (**d**) raw natural rutile (**e**) natural rutile chlorinated for 30 min.

Figure 4 shows the surface morphology of the raw and chlorinated ores. The particle sizes of the raw ilmenite were not significantly different from those of the ilmenite selectively chlorinated for 40 min to remove FeO by mixing coke (comparison between Fig. 4a,b). However, the surface roughness of the synthetic rutile chlorinated for 30 min to produce TiCl_4_ (Fig. 4b) was significantly different from that of the raw natural rutile (Fig. 4d). Kim was investigated that microstructure changes in ilmenite occurring after selective chlorination. In other words, it was noted that the change in the size of the pores due to selective chloride reactions has a significant effect on the response efficiency for TiCl_4_ manufacturing. However, there was no pore comparison with natural rutile^33^. Sohn and Zhou also reported that the chlorination efficiency of the synthetic rutile selectively chlorinated from ilmenite ore was higher than that of natural rutile due to the influence of pores^34^. They, however, did not consider the application of the shrinking-core model, unlike Swanepoel^32^. They mentioned that this was because pore diffusion was partially generated by chemical reactions in the particles. The reaction efficiency of the synthetic rutile with higher porosity due to the removal of FeO (Fig. 4c) was higher than that of the natural rutile (Fig. 4e), and thus chlorination was accelerated for the synthetic rutile. This was consistent with the reaction results of Fig. 3 and the results of Sohn and Zhou (1999).

**Figure 5:**
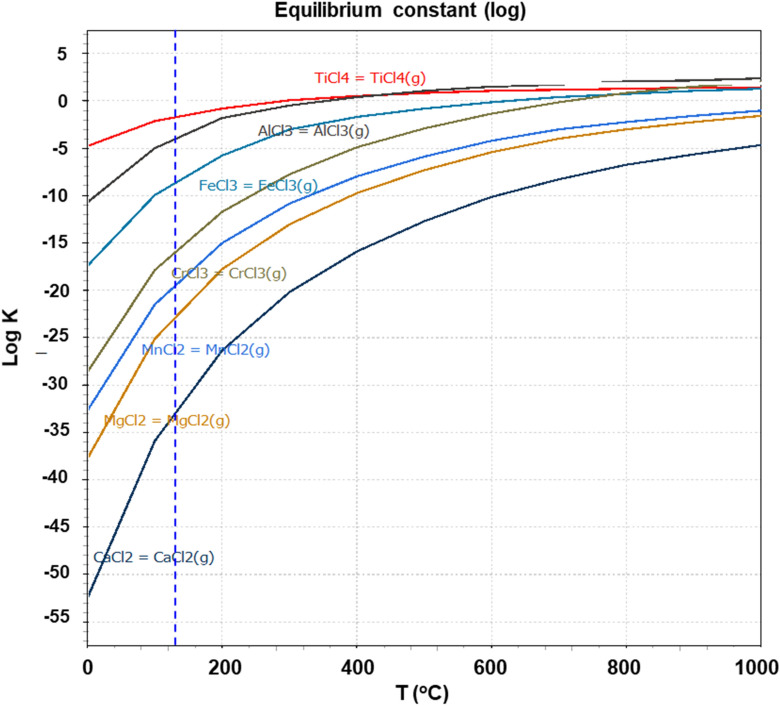
Calculation results of the vapor pressure of TiCl_4_ and residual chloride.

TiCl_4_, manufactured by chloride reaction using synthetic rutile and natural rutile ore, has a trace amount difference depending on the impurities present in the ore. However, all TiCl_4_ manufactured using both ores satisfied 99.9% purity or higher. Also, the difference between the residual impurities TiCl_4_ and the steam pressure is large, so it is judged that there is a possibility of further improvement in purity if the distillation process is carried out using the difference in the steam pressure of each chloride as shown in Fig. 5.

**Figure 6:**
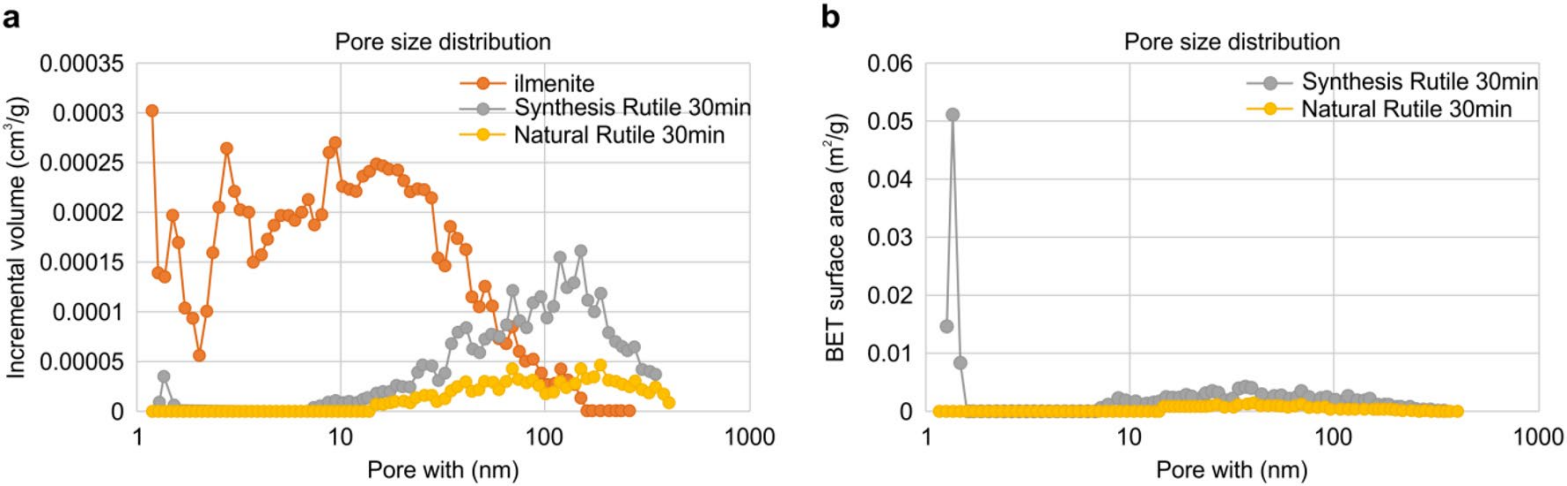
Analysis results of (**a**) incremental volume of ilmenite and synthetic/natural rutiles chlorinated for 30 min (**b**) BET surface area of synthetic/natural rutiles chlorinated for 30 min.

For the quantitative evaluation of the pore formation in the ores, the specific surface area measurement evaluation was conducted, and the results are shown in Fig. 6. As shown in Fig. 6a, unlike the pore size distribution between 1 and 100 nm for the raw ilmenite ore, pore sizes were distributed in the range between 10 and 600 nm for the synthetic rutile selectively chlorinated for 40 min. This indicates that micropores existed in the raw ilmenite ore and that the distribution range of the pores was later significantly increased by the chlorination. The pore size distribution range of the synthetic rutile was similar to that of the natural rutile after chlorination, but the volume of the open pores was larger for the synthetic rutile. As shown in Fig. 6b, the overall surface areas determined by the BET method of the synthetic rutile and natural rutile were 0.98 and 0.14 m^2^/g, respectively, compared to the ilmenite ore with 11.8 m^2^/g. Therefore, when the pore volume and specific surface area distributions were compared, the specific surface area of the synthetic rutile was relatively higher due to the pores with 1–2 nm sizes, indicating that there were more micropores. In the case of the synthetic rutile, the removal of FeO by selective chlorination generated empty spaces in the matrix. It appears that the active input of chlorine into such spaces caused the more active reaction with the remaining TiO_2_ than for chlorination of the natural rutile. The synthetic/natural rutile ores started reaction from the surface, and the slowly decreasing particle sizes were similar as shown in Fig. 3. In terms of the reaction efficiency, however, it was found that more particles were generated, and this appears to be because there were more sites capable of chlorination despite the same rutile types.

**Figure 7:**
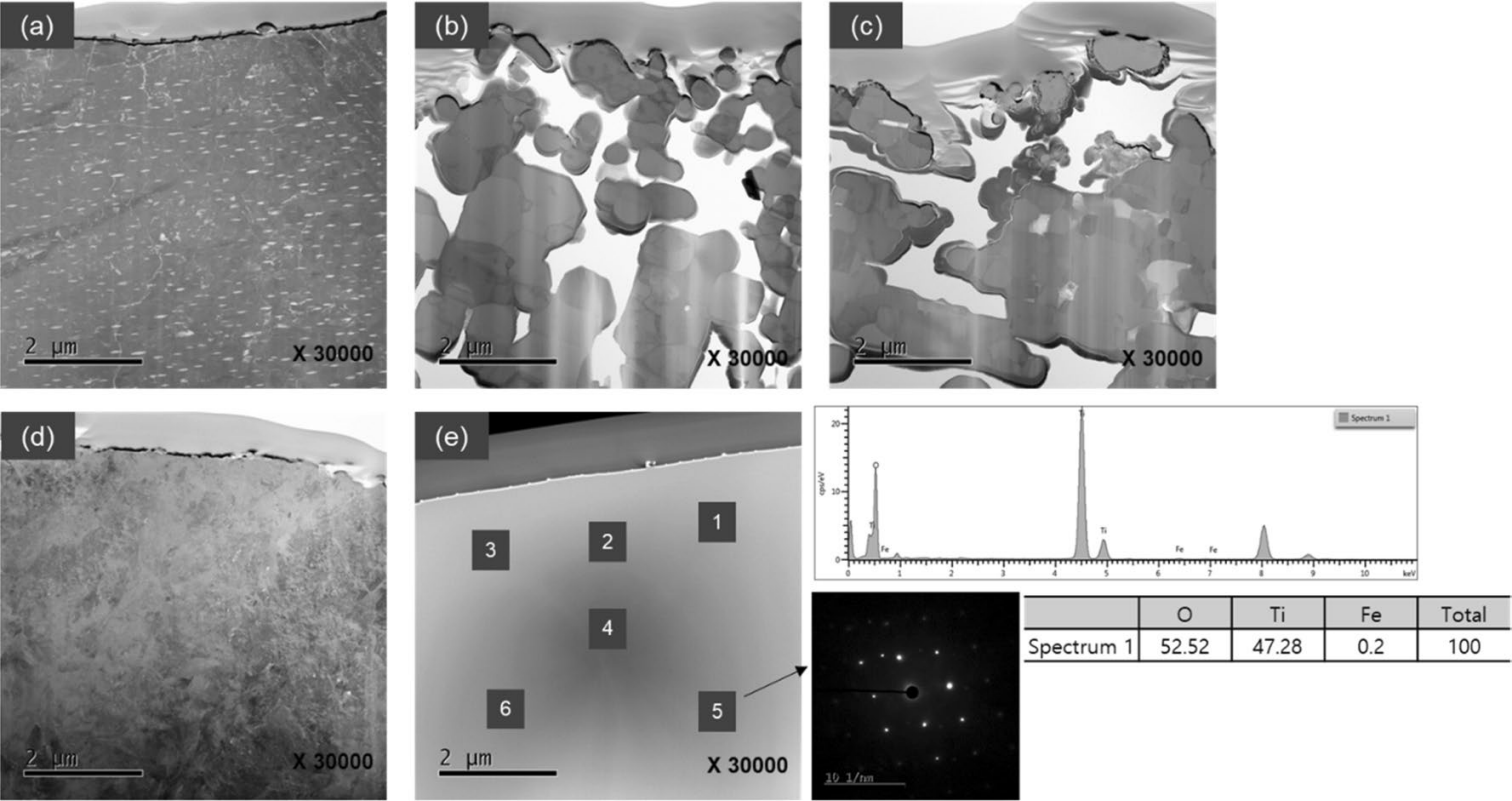
Micrographs of TEM observations for (**a**) raw ilmenite (**b**) ilmenite chlorinated for 40 min (**c**) synthetic rutile chlorinated for 30 min (**d**) raw natural rutile (**e**) natural rutile chlorinated for 30 min.

The TEM micrographs in Fig. 7 show that the porosity generated by the initial selective chlorination was not significantly affected by the production of TiCl_4_, and it appears that most of the shapes of the initial pores were maintained. In the case of the natural rutile, the reaction cross section became stronger. As shown in Fig. 7d, the natural rutile with multi-phase multiple grains was subjected to single crystallization as shown in Fig. 7e because the distribution of crystal phases was summarized during the generation of TiCl_4_. In Fig. 7e, the diffraction pattern (DP) was measured for each section and it was confirmed that each spot had the same TiO_2_ composition. It was also confirmed that the entire area was the grain of the same crystal structure. It is judged that further research is required in the future on the crystal structure and density of natural rutile during chlorination.

## Conclusion

In this study, the chlorination efficiency of synthetic rutile, which was researched by removing FeO_x_ from ilmenite through selective chlorination using coke as a reducing agent and Cl_2_ gas, and natural rutile also was examined for manufacturing of TiCl_4_.

1. For selective chlorination of FeO in ilmenite ore, it was found that the purity and yield of the synthetic rutile were determined by the input amounts of coke and chlorine. It was confirmed that TiCl_4_ was also generated at the same time when the input amount of one mole of ilmenite reacted with more than one mole of chlorine. Therefore, it is very important to improve the reaction efficiency and purity of TiCl_4_ depending on the amount of coke and chlorine gas injected with the titanium ore. In this way, porous synthetic rutile can be obtained depending on the efficiency of the selective chloride process, and can be linked to high purity of TiCl_4_ depending on the purity of the synthetic rutile.
2. As shown by SEM analysis, the synthetic rutile remained porous after chlorination, thereby exhibiting higher reaction efficiency than natural rutile. This is due to the fact that selective elimination of FeO does not reduce the particle size of the ilmenite ore in the manufacture of synthetic rutiles using ilmenite ore, and that there is a pore where FeO has been removed, thereby increasing the reaction efficiency of chlorine gas.
3. The BET measurement results showed that the raw ilmenite ore was dominated by micropores as large as several nanometers, but the pore size increased for the synthetic rutile subjected to selective chlorination for 40 min. However, it was confirmed that more micropores were distributed in the synthetic rutile than in natural rutile, and it was also confirmed that such micropores had a larger influence on the chlorination efficiency.

## References

[1] Zheng H, Okabe TH (2008). Recovery of titanium metal scrap by utilizing chloride wastes. J. Alloys Compd..

[2] Faller K, Froes FH (2001). The use of titanium in family automobiles: Current trends. JOM..

[3] Moriya A, Kanai A (1993). Titanium sponge production at sumitomo sitix corporation. Shigen-to-Sozai..

[4] Powell R (1968). Titanium oxide and Titanium tetrachloride.

[5] Itoh S, Suga T, Yakizawa TH, Nagasaka T (2007). Application of 28 GHz microwave irradiation to oxidation of ilmenite ore for new rutile extraction process. ISIJ Int..

[6] Li C, Liang B, Guo L (2007). Dissolution of mechanically activated Panzhihua ilmenites in dilute solutions of sulphuric acid. Hydrometallurgy.

[7] Hong T, Jung EJ, Lee DH (2019). Segregation of glass beads in three-sectional tapered fluidized beds with ternary system. Powder Technol..

[8] Bracanin BF, Cassidy W, MacKay JM, Hockin HW (1972). The development of a direct reduction and leach process for ilmenite upgrading. AIME-TMS Pap..

[9] Sinha, H. N. Proceedings of the Eleventh Commonwealth Mining and Metallurgical Congress. *Institute of Mining and Metallurgy, London*. 669 (1979).

[10] Becher, R. G. Improved process for the beneficiation of ores containing contaminating iron, *Australian., Patent* 247110 (1963).

[11] Samal S (2013). Preparation of synthetic rutile from pre-treated ilmenite/Ti-rich slag with phenol and resorcinol leaching solutions. Hydrometallurgy.

[12] Samal S (2011). The dissolution of iron in the hydrochloric acid leach of titania slag obtained from plasma melt separation of metalized ilmenite. Chem. Eng. Res. Des..

[13] Li K (2020). Investigations on the microwave absorption properties and thermal behavior of vanadium slag: Improvement in microwave oxidation roasting for recycling vanadium and chromium. J. Hazard. Mater..

[14] Chen G (2020). Simultaneous removal of Cr(III) and V(V) and enhanced synthesis of high-grade rutile TiO_2_ based on sodium carbonate decomposition. J. Hazard. Mater..

[15] Kang J (2020). Synthesis of rutile TiO_2_ powder by microwave-enhanced roasting followed by hydrochloric acid leaching. Adv. Powder. Tech..

[16] Natziger, R. H., Elger, G. W. Preparation of titanium feedstock from Minnesota Ilmenite by smelting and sulfation-leaching. *US Bureau of Mines. Report Invest* No. 9065 (1987).

[17] Hope, M. Mineral sand price forecasts. *Diversified Metals & Mining, Asia Pacific/Australia* (2016).

[18] Palliyaguru L (2020). A simple and novel synthetic route to prepare anatase TiO_2_ nanopowders from natural ilmenite via the H_3_PO_4_/NH_3_ process. Int. J. Miner. Metall. Mater..

[19] Wu S (2020). High Cr(VI) adsorption capacity of Rutile titania prepared by hydrolysis of TiCl_4_ with AlCl3. Int. J. Miner. Metall. Mater..

[20] Ma J, Fu G, Zhu M (2020). Influence of TiO_2_ on the melting property and viscosity of Cr-containing high-Ti melting slag. Int. J. Miner. Metall. Mater..

[21] Wen C, Yu Y (1966). A generalized method for pre- diction of the minimum fluidization velocity. Aiche J..

[22] Meinhold G (2010). Rutile and its applications in earth sciences. Earth-Sci. Rev..

[23] Murakami T (2013). Quantitative analysis on contribution of direct reduction of iron oxide in carbon composite. ISIJ Int..

[24] Prakash S (1996). Reduction and sintering of fluxed iron ore pellets: A comprehensive review. J. S. Afr. Inst. Min. Metall.

[25] Huang Z, Yi L, Jiang T (2012). Mechanisms of strength decrease in the initial reduction of iron ore oxide pellets. Powder Technol..

[26] Pineau A, Kanari N, Gaballah I (2006). Kinetics of reduction of iron oxides by H_2_: Part I: Low temperature reduction of hematite. Thermochim. Acta.

[27] Gan M (2018). Preparing high-strength titanium pellets for ironmaking as furnace protector: Optimum route for ilmenite oxidation and consolidation. Powder Technol..

[28] Fu X, Wang Y, Wei F (2010). Phase transitions and reaction mechanism of ilmenite oxidation. Metall. Mater. Trans. A.

[29] Allen N (2003). Effect of roasting temperature on the magnetism of ilmenite. Phys. Sep. Sci. Eng..

[30] Kang J, Okabe TH (2014). Thermodynamic consideration of the removal of iron from titanium ore by selective chlorination. Metall. Mater. Trans. B.

[31] Kang J, Okabe TH (2014). Production of titanium dioxide directly from titanium ore through selective chlorination using titanium tetrachloride. Mater. Trans..

[32] Swanepoel, J. J. Process development for the removal of iron from nitride ilmenite. *M. Eng. Thesis, University of Pretoria* (2010).

[33] Kim J, Lee MS, Jung EJ (2020). A study of formation behavior of porous structure induced by selective chlorination of ilmenite. Mater. Chem. Phys.

[34] Sohn HY, Zhou L (1999). The chlorination kinetics of beneficiated ilmenite particles by CO+Cl_2_ mixtures. Chem. Eng. J..

